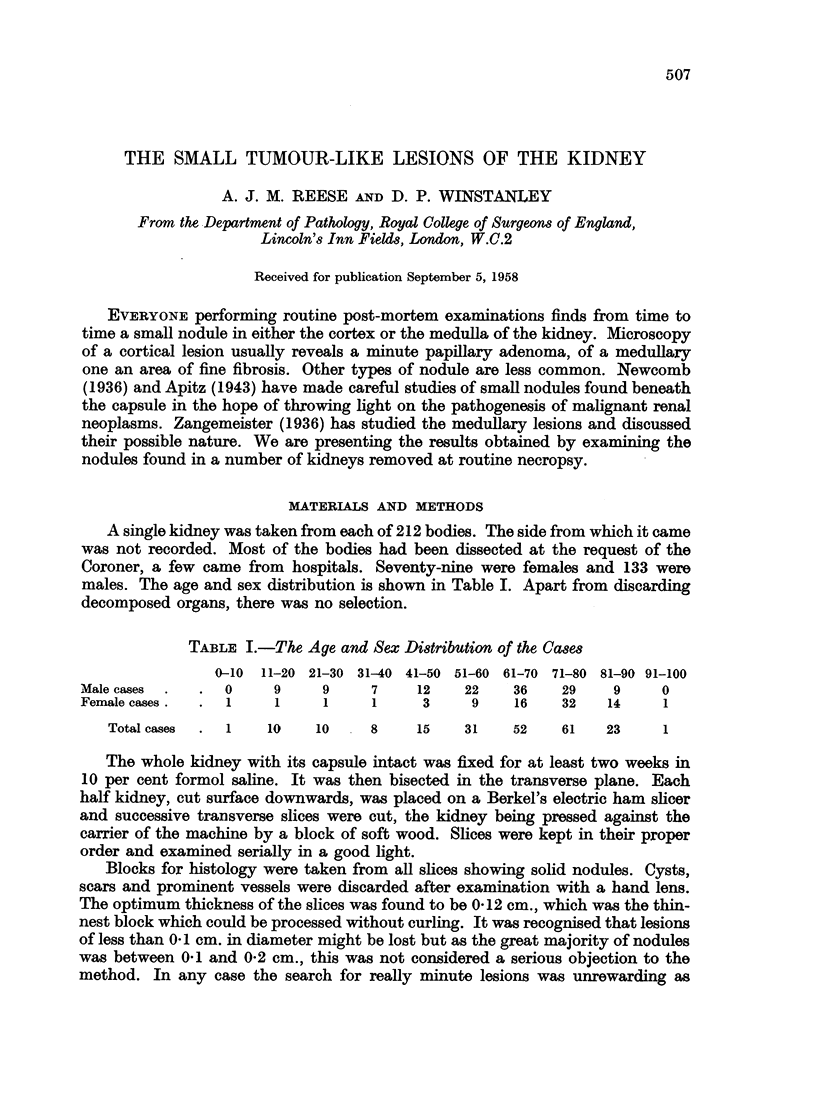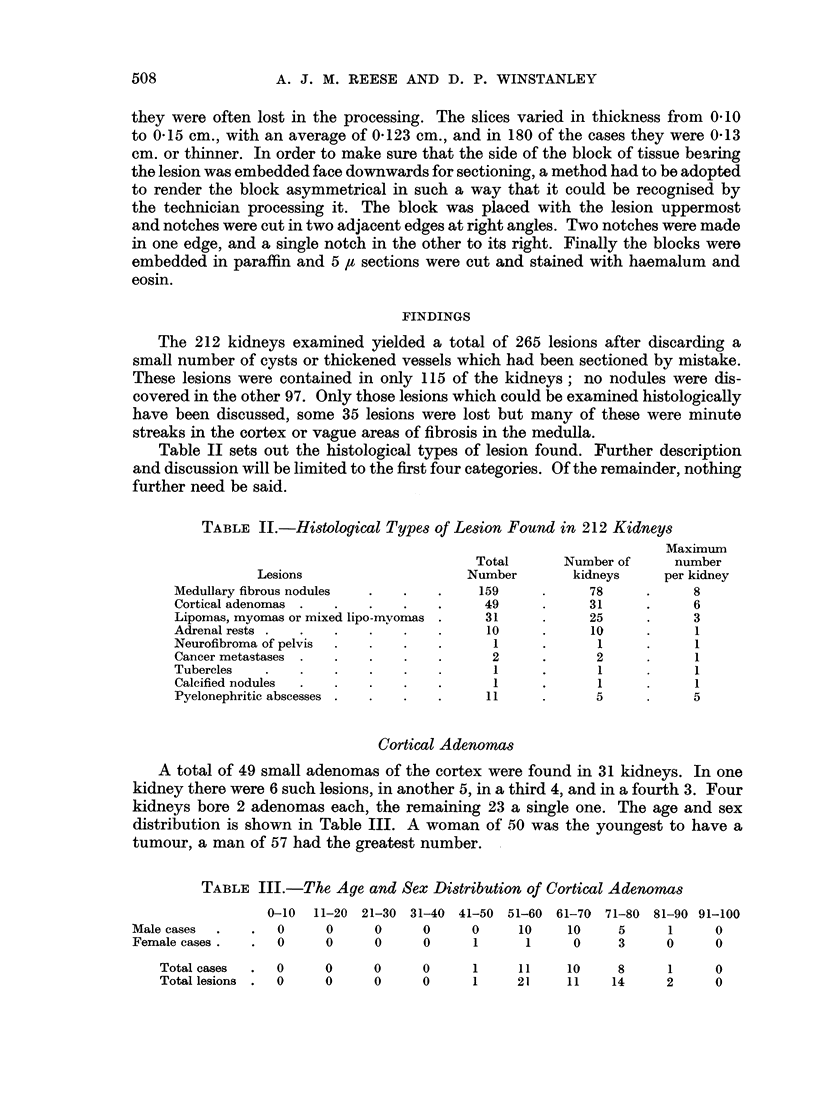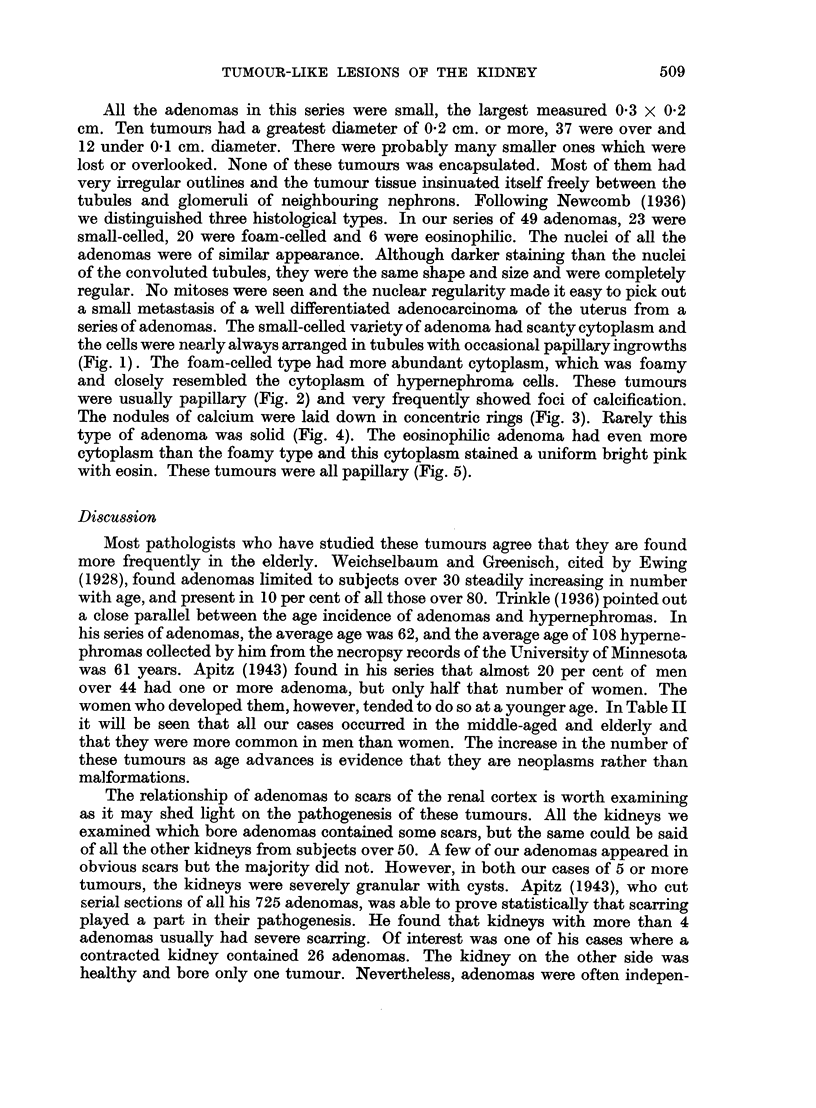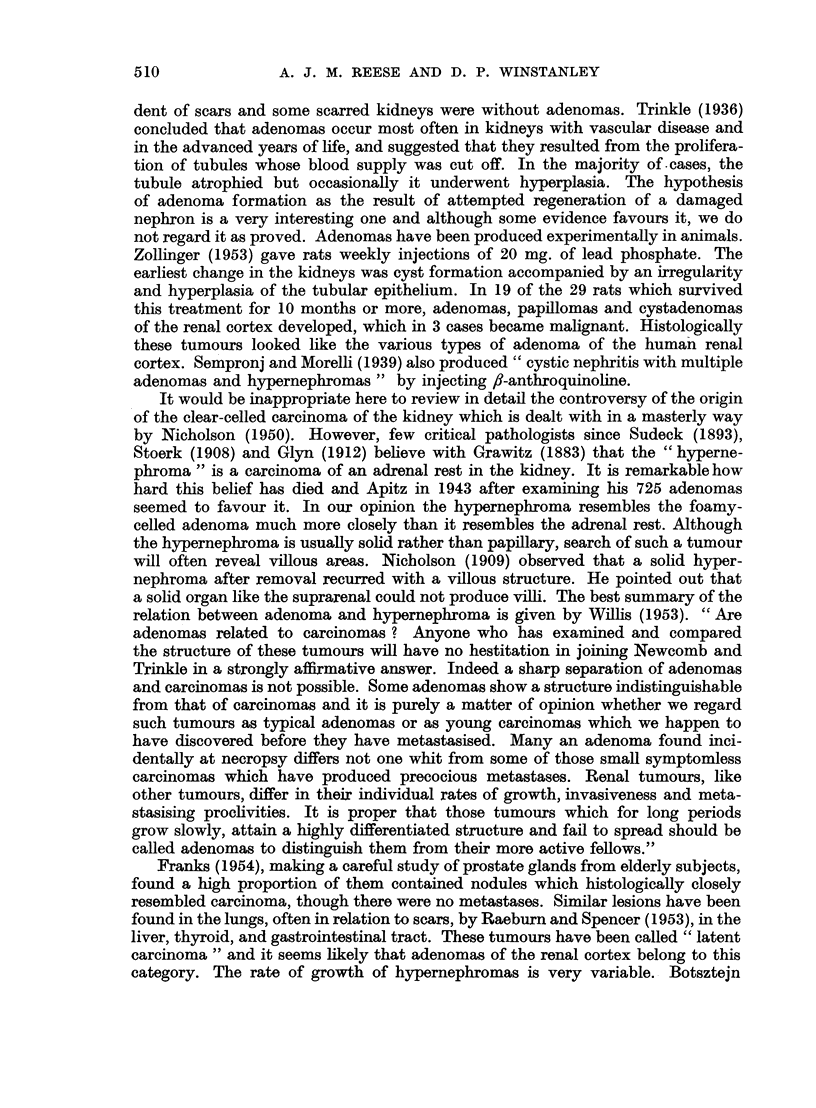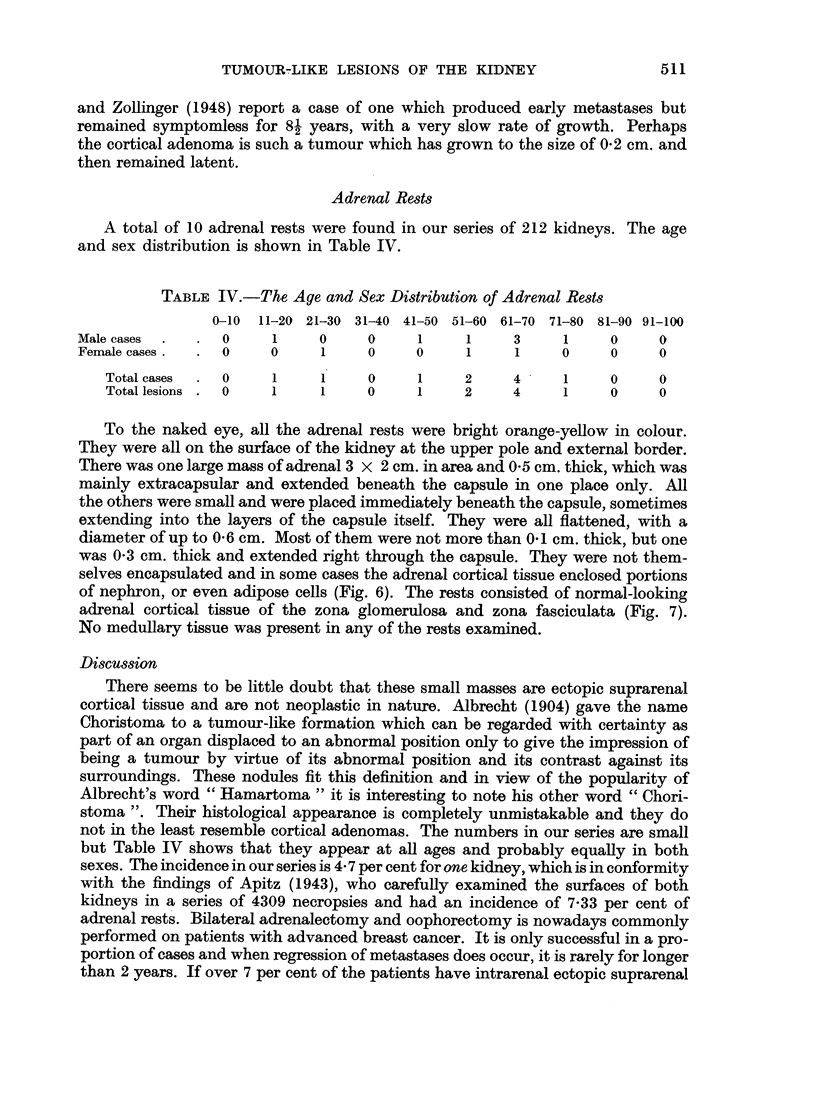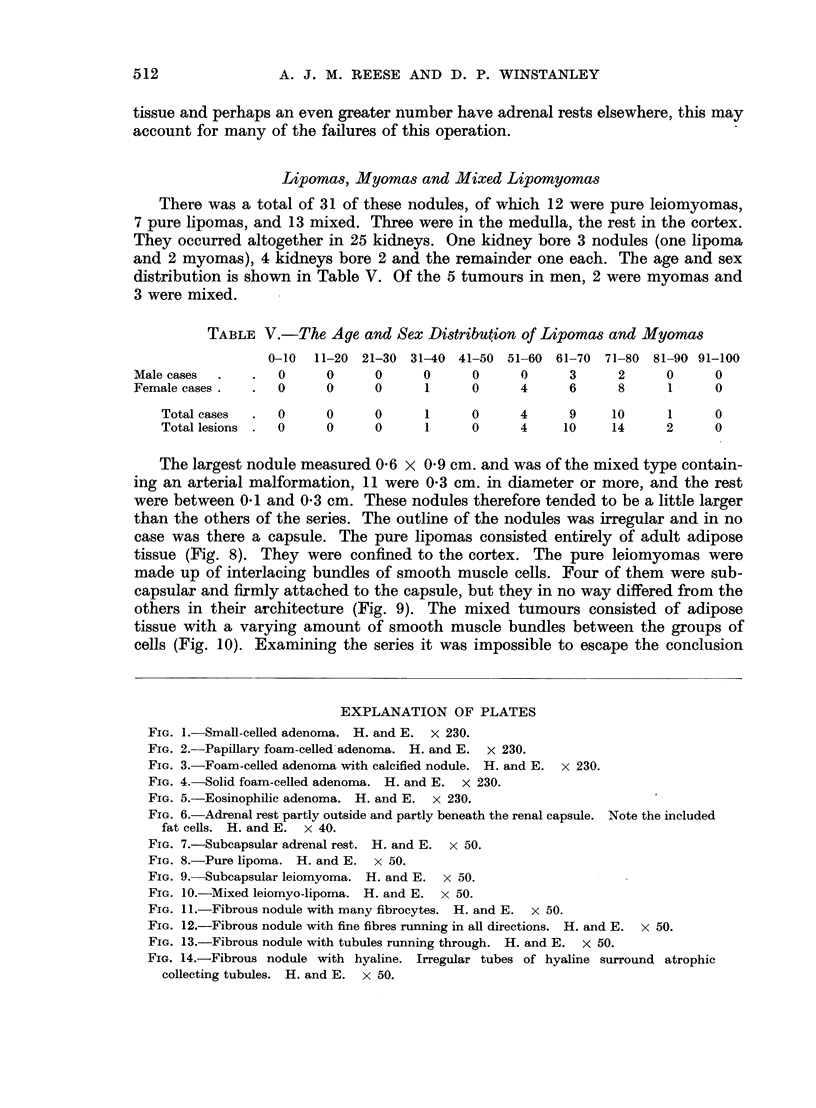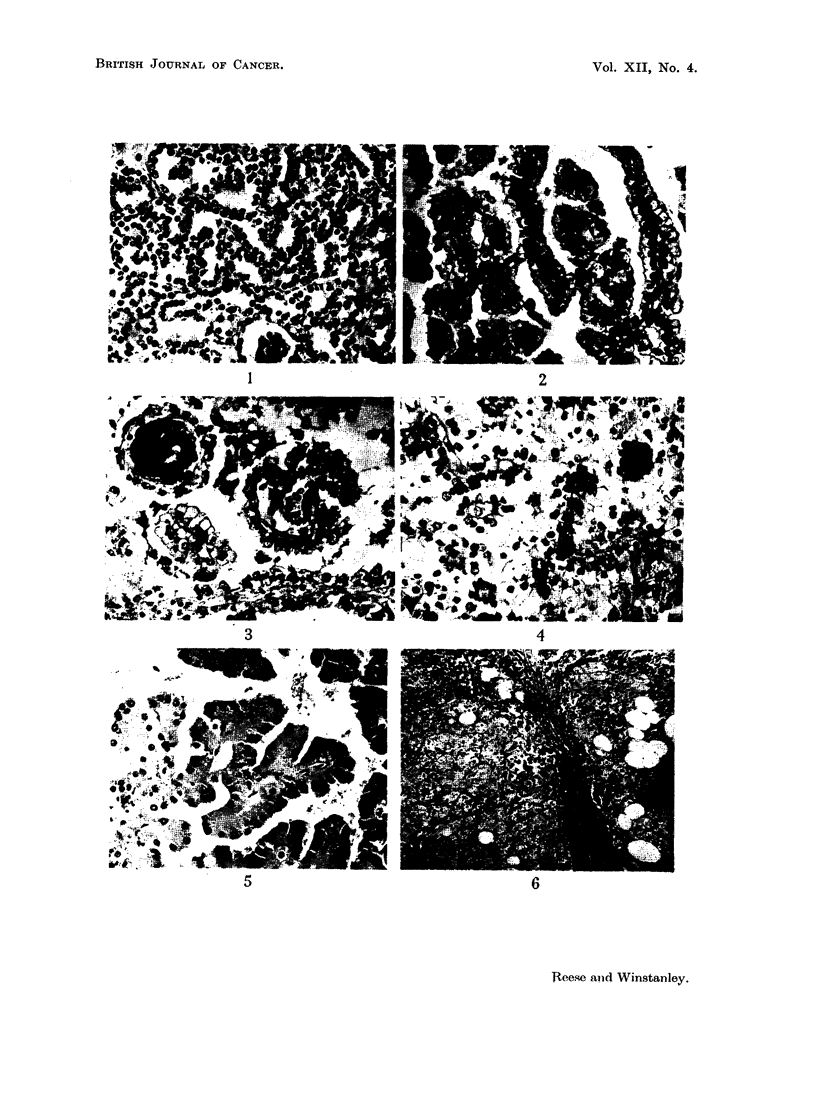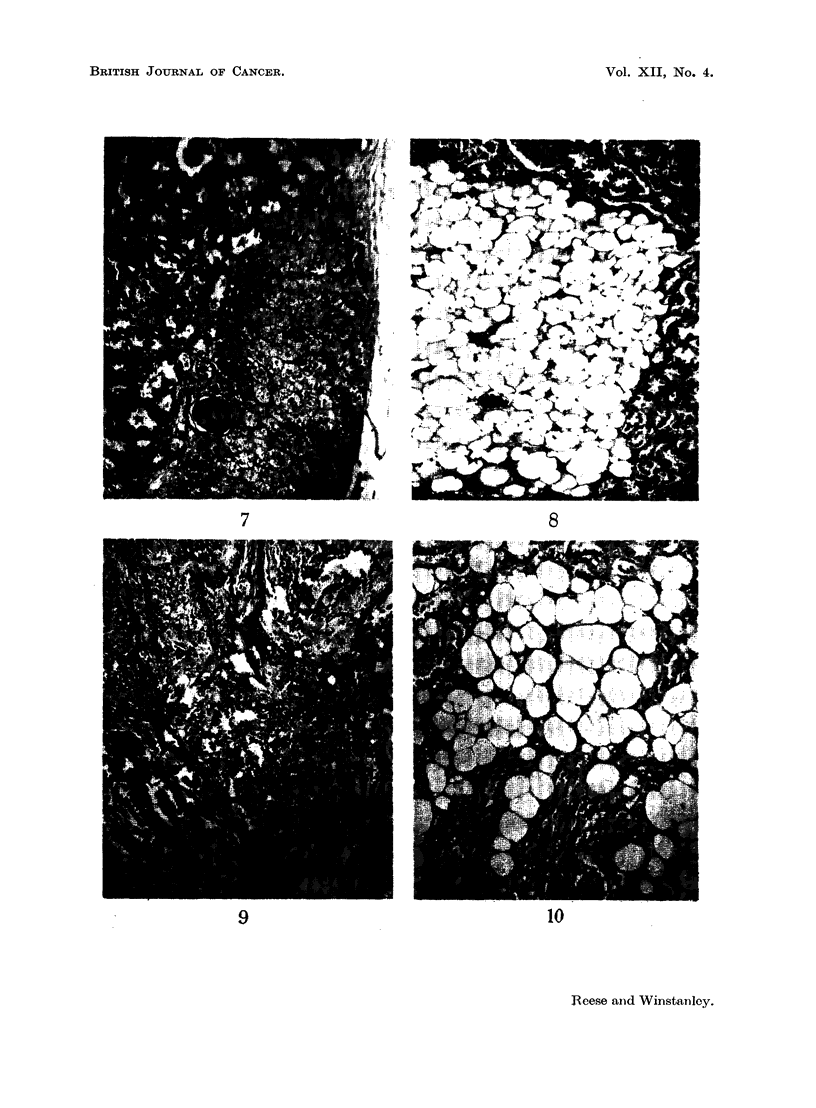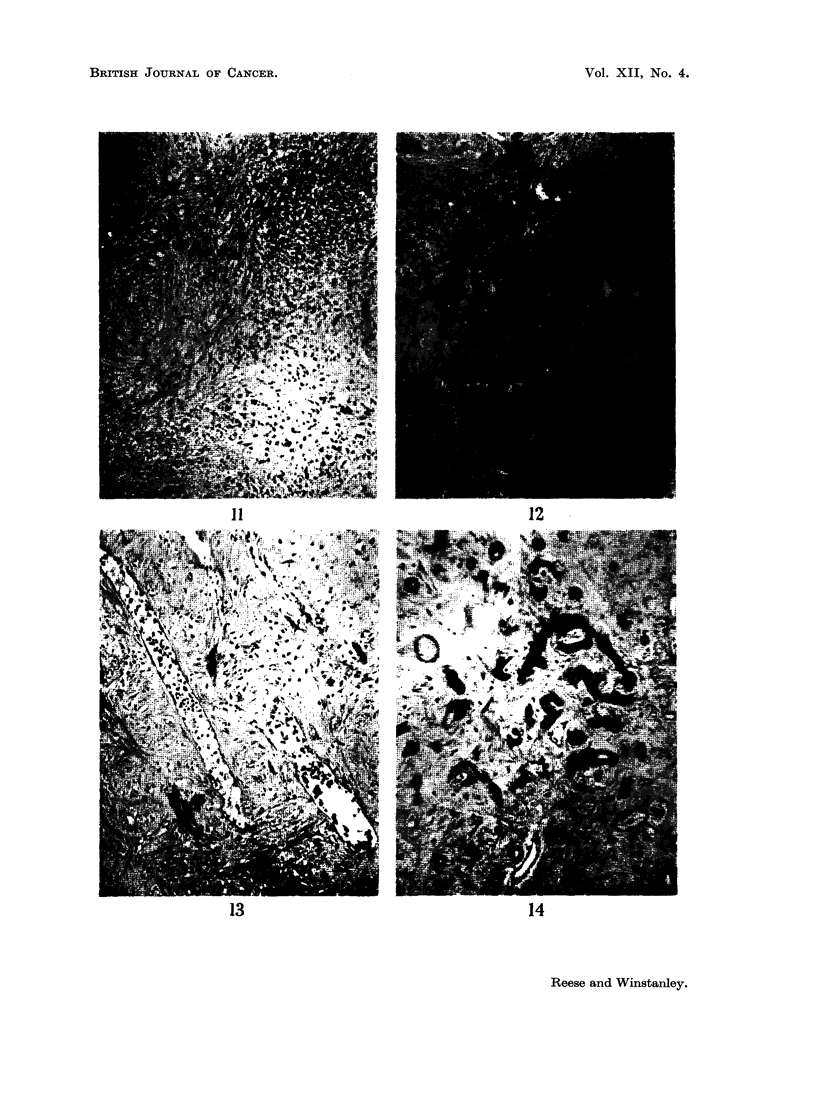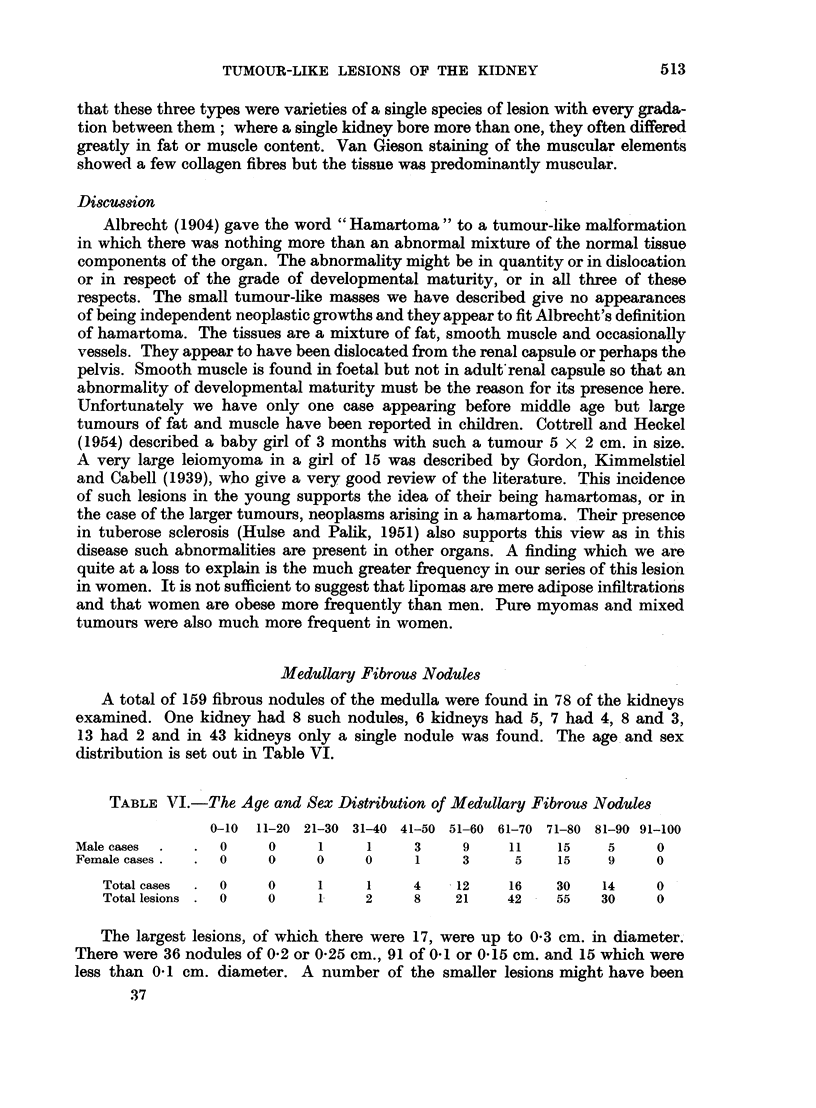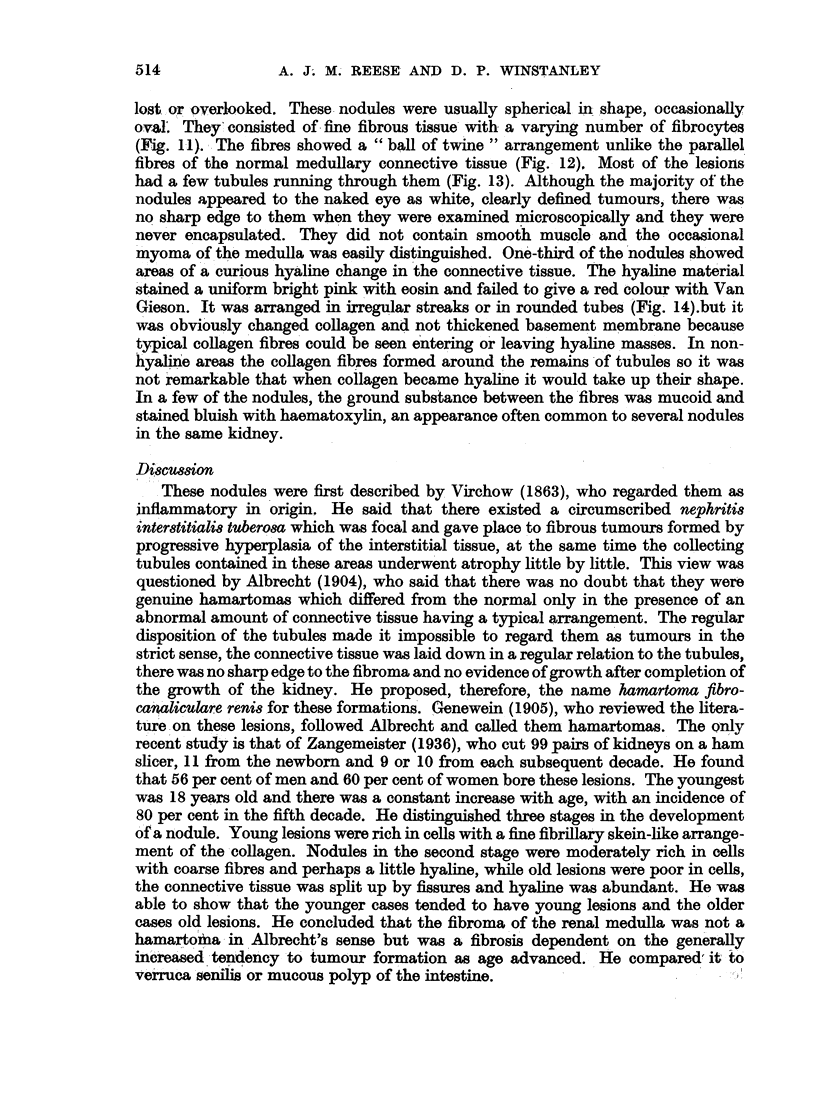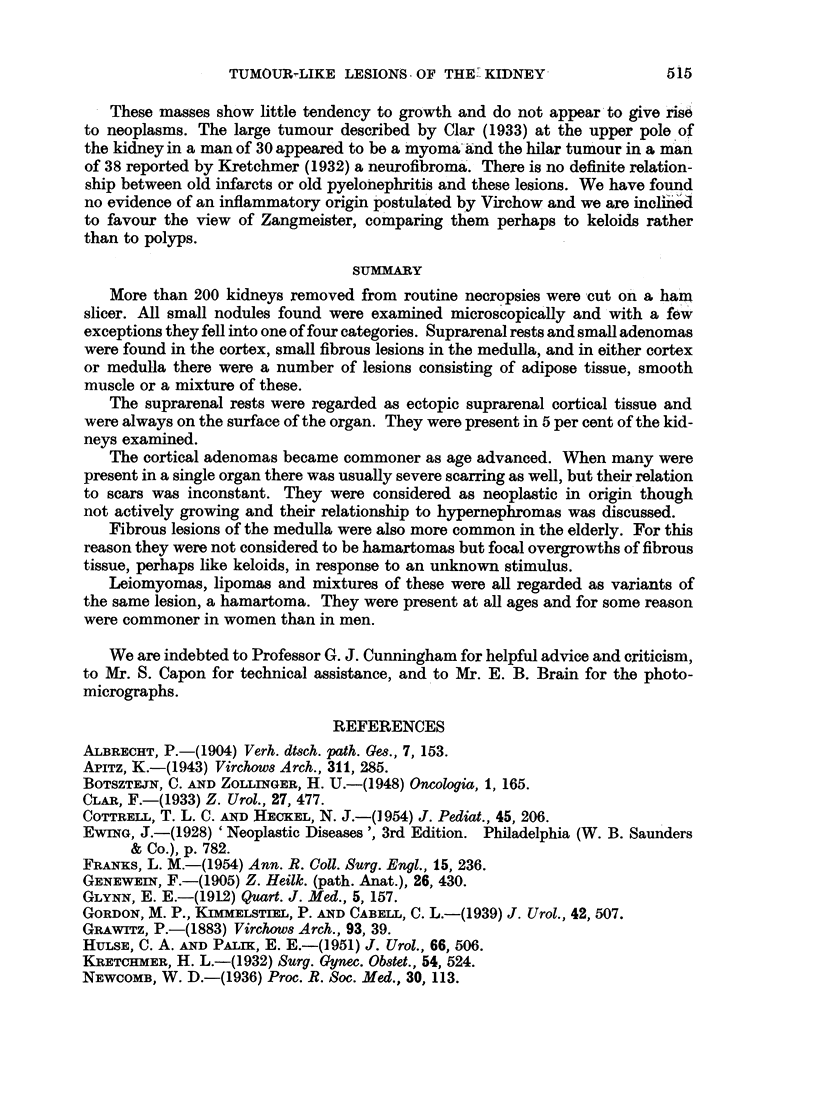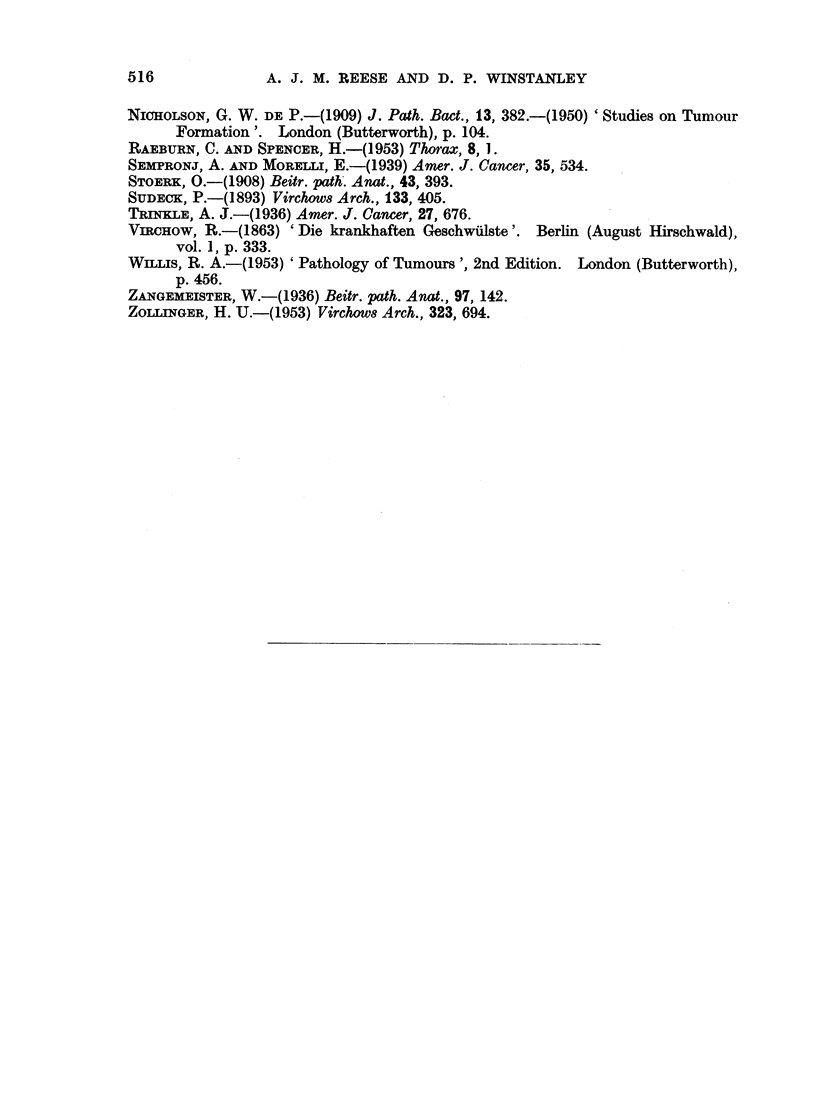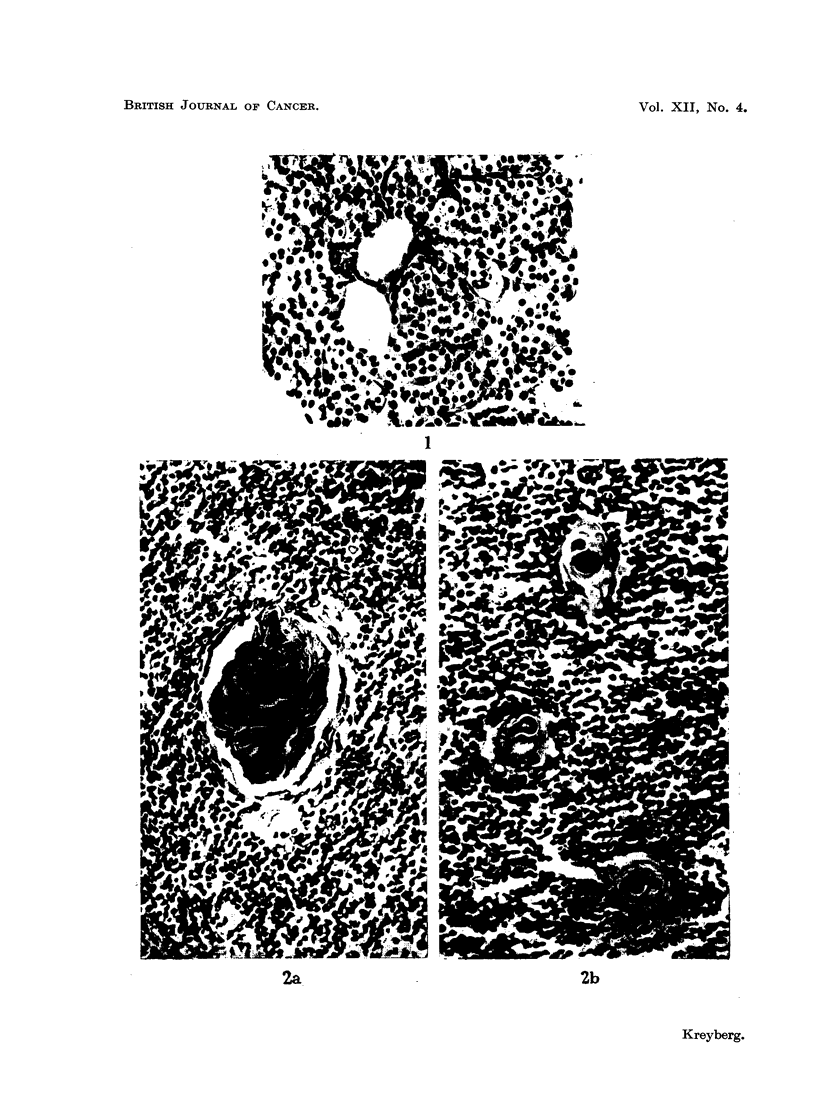# The Small Tumour-like Lesions of the Kidney

**DOI:** 10.1038/bjc.1958.58

**Published:** 1958-12

**Authors:** A. J. M. Reese, D. P. Winstanley

## Abstract

**Images:**


### THE SMALL TUMOUR-LIKE LESIONS OF THE KIDNEY

A. J. M. REESE AND D. P. WINSTANLEY

*From the Department of Pathology, Royal College of Surgeons of England, Lincoln's Inn Fields, London, W.C.2*

Received for publication September 5, 1958

EVERYONE performing routine post-mortem examinations finds from time to time a small nodule in either the cortex or the medulla of the kidney. Microscopy of a cortical lesion usually reveals a minute papillary adenoma, of a medullary one an area of fine fibrosis. Other types of nodule are less common. Newcomb (1936) and Apitz (1943) have made careful studies of small nodules found beneath the capsule in the hope of throwing light on the pathogenesis of malignant renal neoplasms. Zangemeister (1936) has studied the medullary lesions and discussed their possible nature. We are presenting the results obtained by examining the nodules found in a number of kidneys removed at routine necropsy.

### MATERIALS AND METHODS

A single kidney was taken from each of 212 bodies. The side from which it came was not recorded. Most of the bodies had been dissected at the request of the Coroner, a few came from hospitals. Seventy-nine were females and 133 were males. The age and sex distribution is shown in Table I. Apart from discarding decomposed organs, there was no selection.

TABLE I.—*The Age and Sex Distribution of the Cases*

| | 0–10 | 11–20 | 21–30 | 31–40 | 41–50 | 51–60 | 61–70 | 71–80 | 81–90 | 91–100 |
|---|---|---|---|---|---|---|---|---|---|---|
| Male cases | 0 | 9 | 9 | 7 | 12 | 22 | 36 | 29 | 9 | 0 |
| Female cases | 1 | 1 | 1 | 1 | 3 | 9 | 16 | 32 | 14 | 1 |
| Total cases | 1 | 10 | 10 | 8 | 15 | 31 | 52 | 61 | 23 | 1 |

The whole kidney with its capsule intact was fixed for at least two weeks in 10 per cent formol saline. It was then bisected in the transverse plane. Each half kidney, cut surface downwards, was placed on a Berkel's electric ham slicer and successive transverse slices were cut, the kidney being pressed against the carrier of the machine by a block of soft wood. Slices were kept in their proper order and examined serially in a good light.

Blocks for histology were taken from all slices showing solid nodules. Cysts, scars and prominent vessels were discarded after examination with a hand lens. The optimum thickness of the slices was found to be 0·12 cm., which was the thinnest block which could be processed without curling. It was recognised that lesions of less than 0·1 cm. in diameter might be lost but as the great majority of nodules was between 0·1 and 0·2 cm., this was not considered a serious objection to the method. In any case the search for really minute lesions was unrewarding as they were often lost in the processing. The slices varied in thickness from 0·10 to 0·15 cm., with an average of 0·123 cm., and in 180 of the cases they were 0·13 cm. or thinner. In order to make sure that the side of the block of tissue bearing the lesion was embedded face downwards for sectioning, a method had to be adopted to render the block asymmetrical in such a way that it could be recognised by the technician processing it. The block was placed with the lesion uppermost and notches were cut in two adjacent edges at right angles. Two notches were made in one edge, and a single notch in the other to its right. Finally the blocks were embedded in paraffin and 5 $\mu$ sections were cut and stained with haemalum and eosin.

### FINDINGS

The 212 kidneys examined yielded a total of 265 lesions after discarding a small number of cysts or thickened vessels which had been sectioned by mistake. These lesions were contained in only 115 of the kidneys ; no nodules were discovered in the other 97. Only those lesions which could be examined histologically have been discussed, some 35 lesions were lost but many of these were minute streaks in the cortex or vague areas of fibrosis in the medulla.

Table II sets out the histological types of lesion found. Further description and discussion will be limited to the first four categories. Of the remainder, nothing further need be said.

TABLE II.—*Histological Types of Lesion Found in 212 Kidneys*

| Lesions | Total Number | Number of kidneys | Maximum number per kidney |
|---|---|---|---|
| Medullary fibrous nodules | 159 | 78 | 8 |
| Cortical adenomas | 49 | 31 | 6 |
| Lipomas, myomas or mixed lipo-myomas | 31 | 25 | 3 |
| Adrenal rests | 10 | 10 | 1 |
| Neurofibroma of pelvis | 1 | 1 | 1 |
| Cancer metastases | 2 | 2 | 1 |
| Tubercles | 1 | 1 | 1 |
| Calcified nodules | 1 | 1 | 1 |
| Pyelonephritic abscesses | 11 | 5 | 5 |

### *Cortical Adenomas*

A total of 49 small adenomas of the cortex were found in 31 kidneys. In one kidney there were 6 such lesions, in another 5, in a third 4, and in a fourth 3. Four kidneys bore 2 adenomas each, the remaining 23 a single one. The age and sex distribution is shown in Table III. A woman of 50 was the youngest to have a tumour, a man of 57 had the greatest number.

TABLE III.—*The Age and Sex Distribution of Cortical Adenomas*

| | 0–10 | 11–20 | 21–30 | 31–40 | 41–50 | 51–60 | 61–70 | 71–80 | 81–90 | 91–100 |
|---|---|---|---|---|---|---|---|---|---|---|
| Male cases | 0 | 0 | 0 | 0 | 0 | 10 | 10 | 5 | 1 | 0 |
| Female cases | 0 | 0 | 0 | 0 | 1 | 1 | 0 | 3 | 0 | 0 |
| Total cases | 0 | 0 | 0 | 0 | 1 | 11 | 10 | 8 | 1 | 0 |
| Total lesions | 0 | 0 | 0 | 0 | 1 | 21 | 11 | 14 | 2 | 0 |

All the adenomas in this series were small, the largest measured 0·3 × 0·2 cm. Ten tumours had a greatest diameter of 0·2 cm. or more, 37 were over and 12 under 0·1 cm. diameter. There were probably many smaller ones which were lost or overlooked. None of these tumours was encapsulated. Most of them had very irregular outlines and the tumour tissue insinuated itself freely between the tubules and glomeruli of neighbouring nephrons. Following Newcomb (1936) we distinguished three histological types. In our series of 49 adenomas, 23 were small-celled, 20 were foam-celled and 6 were eosinophilic. The nuclei of all the adenomas were of similar appearance. Although darker staining than the nuclei of the convoluted tubules, they were the same shape and size and were completely regular. No mitoses were seen and the nuclear regularity made it easy to pick out a small metastasis of a well differentiated adenocarcinoma of the uterus from a series of adenomas. The small-celled variety of adenoma had scanty cytoplasm and the cells were nearly always arranged in tubules with occasional papillary ingrowths (Fig. 1). The foam-celled type had more abundant cytoplasm, which was foamy and closely resembled the cytoplasm of hypernephroma cells. These tumours were usually papillary (Fig. 2) and very frequently showed foci of calcification. The nodules of calcium were laid down in concentric rings (Fig. 3). Rarely this type of adenoma was solid (Fig. 4). The eosinophilic adenoma had even more cytoplasm than the foamy type and this cytoplasm stained a uniform bright pink with eosin. These tumours were all papillary (Fig. 5).

*Discussion*

Most pathologists who have studied these tumours agree that they are found more frequently in the elderly. Weichselbaum and Greenisch, cited by Ewing (1928), found adenomas limited to subjects over 30 steadily increasing in number with age, and present in 10 per cent of all those over 80. Trinkle (1936) pointed out a close parallel between the age incidence of adenomas and hypernephromas. In his series of adenomas, the average age was 62, and the average age of 108 hypernephromas collected by him from the necropsy records of the University of Minnesota was 61 years. Apitz (1943) found in his series that almost 20 per cent of men over 44 had one or more adenoma, but only half that number of women. The women who developed them, however, tended to do so at a younger age. In Table II it will be seen that all our cases occurred in the middle-aged and elderly and that they were more common in men than women. The increase in the number of these tumours as age advances is evidence that they are neoplasms rather than malformations.

The relationship of adenomas to scars of the renal cortex is worth examining as it may shed light on the pathogenesis of these tumours. All the kidneys we examined which bore adenomas contained some scars, but the same could be said of all the other kidneys from subjects over 50. A few of our adenomas appeared in obvious scars but the majority did not. However, in both our cases of 5 or more tumours, the kidneys were severely granular with cysts. Apitz (1943), who cut serial sections of all his 725 adenomas, was able to prove statistically that scarring played a part in their pathogenesis. He found that kidneys with more than 4 adenomas usually had severe scarring. Of interest was one of his cases where a contracted kidney contained 26 adenomas. The kidney on the other side was healthy and bore only one tumour. Nevertheless, adenomas were often independent of scars and some scarred kidneys were without adenomas. Trinkle (1936) concluded that adenomas occur most often in kidneys with vascular disease and in the advanced years of life, and suggested that they resulted from the proliferation of tubules whose blood supply was cut off. In the majority of cases, the tubule atrophied but occasionally it underwent hyperplasia. The hypothesis of adenoma formation as the result of attempted regeneration of a damaged nephron is a very interesting one and although some evidence favours it, we do not regard it as proved. Adenomas have been produced experimentally in animals. Zollinger (1953) gave rats weekly injections of 20 mg. of lead phosphate. The earliest change in the kidneys was cyst formation accompanied by an irregularity and hyperplasia of the tubular epithelium. In 19 of the 29 rats which survived this treatment for 10 months or more, adenomas, papillomas and cystadenomas of the renal cortex developed, which in 3 cases became malignant. Histologically these tumours looked like the various types of adenoma of the human renal cortex. Sempronj and Morelli (1939) also produced " cystic nephritis with multiple adenomas and hypernephromas " by injecting $\beta$-anthroquinoline.

It would be inappropriate here to review in detail the controversy of the origin of the clear-celled carcinoma of the kidney which is dealt with in a masterly way by Nicholson (1950). However, few critical pathologists since Sudeck (1893), Stoerk (1908) and Glyn (1912) believe with Grawitz (1883) that the " hypernephroma " is a carcinoma of an adrenal rest in the kidney. It is remarkable how hard this belief has died and Apitz in 1943 after examining his 725 adenomas seemed to favour it. In our opinion the hypernephroma resembles the foamy-celled adenoma much more closely than it resembles the adrenal rest. Although the hypernephroma is usually solid rather than papillary, search of such a tumour will often reveal villous areas. Nicholson (1909) observed that a solid hypernephroma after removal recurred with a villous structure. He pointed out that a solid organ like the suprarenal could not produce villi. The best summary of the relation between adenoma and hypernephroma is given by Willis (1953). " Are adenomas related to carcinomas ? Anyone who has examined and compared the structure of these tumours will have no hestitation in joining Newcomb and Trinkle in a strongly affirmative answer. Indeed a sharp separation of adenomas and carcinomas is not possible. Some adenomas show a structure indistinguishable from that of carcinomas and it is purely a matter of opinion whether we regard such tumours as typical adenomas or as young carcinomas which we happen to have discovered before they have metastasised. Many an adenoma found incidentally at necropsy differs not one whit from some of those small symptomless carcinomas which have produced precocious metastases. Renal tumours, like other tumours, differ in their individual rates of growth, invasiveness and metastasising proclivities. It is proper that those tumours which for long periods grow slowly, attain a highly differentiated structure and fail to spread should be called adenomas to distinguish them from their more active fellows."

Franks (1954), making a careful study of prostate glands from elderly subjects, found a high proportion of them contained nodules which histologically closely resembled carcinoma, though there were no metastases. Similar lesions have been found in the lungs, often in relation to scars, by Raeburn and Spencer (1953), in the liver, thyroid, and gastrointestinal tract. These tumours have been called " latent carcinoma " and it seems likely that adenomas of the renal cortex belong to this category. The rate of growth of hypernephromas is very variable. Botsztejn and Zollinger (1948) report a case of one which produced early metastases but remained symptomless for 8½ years, with a very slow rate of growth. Perhaps the cortical adenoma is such a tumour which has grown to the size of 0·2 cm. and then remained latent.

### *Adrenal Rests*

A total of 10 adrenal rests were found in our series of 212 kidneys. The age and sex distribution is shown in Table IV.

TABLE IV.—*The Age and Sex Distribution of Adrenal Rests*

| | 0–10 | 11–20 | 21–30 | 31–40 | 41–50 | 51–60 | 61–70 | 71–80 | 81–90 | 91–100 |
|---|---|---|---|---|---|---|---|---|---|---|
| Male cases | 0 | 1 | 0 | 0 | 1 | 1 | 3 | 1 | 0 | 0 |
| Female cases | 0 | 0 | 1 | 0 | 0 | 1 | 1 | 0 | 0 | 0 |
| Total cases | 0 | 1 | 1 | 0 | 1 | 2 | 4 | 1 | 0 | 0 |
| Total lesions | 0 | 1 | 1 | 0 | 1 | 2 | 4 | 1 | 0 | 0 |

To the naked eye, all the adrenal rests were bright orange-yellow in colour. They were all on the surface of the kidney at the upper pole and external border. There was one large mass of adrenal 3 × 2 cm. in area and 0·5 cm. thick, which was mainly extracapsular and extended beneath the capsule in one place only. All the others were small and were placed immediately beneath the capsule, sometimes extending into the layers of the capsule itself. They were all flattened, with a diameter of up to 0·6 cm. Most of them were not more than 0·1 cm. thick, but one was 0·3 cm. thick and extended right through the capsule. They were not themselves encapsulated and in some cases the adrenal cortical tissue enclosed portions of nephron, or even adipose cells (Fig. 6). The rests consisted of normal-looking adrenal cortical tissue of the zona glomerulosa and zona fasciculata (Fig. 7). No medullary tissue was present in any of the rests examined.

#### *Discussion*

There seems to be little doubt that these small masses are ectopic suprarenal cortical tissue and are not neoplastic in nature. Albrecht (1904) gave the name Choristoma to a tumour-like formation which can be regarded with certainty as part of an organ displaced to an abnormal position only to give the impression of being a tumour by virtue of its abnormal position and its contrast against its surroundings. These nodules fit this definition and in view of the popularity of Albrecht's word " Hamartoma " it is interesting to note his other word " Choristoma ". Their histological appearance is completely unmistakable and they do not in the least resemble cortical adenomas. The numbers in our series are small but Table IV shows that they appear at all ages and probably equally in both sexes. The incidence in our series is 4·7 per cent for *one* kidney, which is in conformity with the findings of Apitz (1943), who carefully examined the surfaces of both kidneys in a series of 4309 necropsies and had an incidence of 7·33 per cent of adrenal rests. Bilateral adrenalectomy and oophorectomy is nowadays commonly performed on patients with advanced breast cancer. It is only successful in a proportion of cases and when regression of metastases does occur, it is rarely for longer than 2 years. If over 7 per cent of the patients have intrarenal ectopic suprarenal tissue and perhaps an even greater number have adrenal rests elsewhere, this may account for many of the failures of this operation.

### *Lipomas, Myomas and Mixed Lipomyomas*

There was a total of 31 of these nodules, of which 12 were pure leiomyomas, 7 pure lipomas, and 13 mixed. Three were in the medulla, the rest in the cortex. They occurred altogether in 25 kidneys. One kidney bore 3 nodules (one lipoma and 2 myomas), 4 kidneys bore 2 and the remainder one each. The age and sex distribution is shown in Table V. Of the 5 tumours in men, 2 were myomas and 3 were mixed.

TABLE V.—*The Age and Sex Distribution of Lipomas and Myomas*

| | 0–10 | 11–20 | 21–30 | 31–40 | 41–50 | 51–60 | 61–70 | 71–80 | 81–90 | 91–100 |
|---|---|---|---|---|---|---|---|---|---|---|
| Male cases | 0 | 0 | 0 | 0 | 0 | 0 | 3 | 2 | 0 | 0 |
| Female cases | 0 | 0 | 0 | 1 | 0 | 4 | 6 | 8 | 1 | 0 |
| Total cases | 0 | 0 | 0 | 1 | 0 | 4 | 9 | 10 | 1 | 0 |
| Total lesions | 0 | 0 | 0 | 1 | 0 | 4 | 10 | 14 | 2 | 0 |

The largest nodule measured 0·6 × 0·9 cm. and was of the mixed type containing an arterial malformation, 11 were 0·3 cm. in diameter or more, and the rest were between 0·1 and 0·3 cm. These nodules therefore tended to be a little larger than the others of the series. The outline of the nodules was irregular and in no case was there a capsule. The pure lipomas consisted entirely of adult adipose tissue (Fig. 8). They were confined to the cortex. The pure leiomyomas were made up of interlacing bundles of smooth muscle cells. Four of them were subcapsular and firmly attached to the capsule, but they in no way differed from the others in their architecture (Fig. 9). The mixed tumours consisted of adipose tissue with a varying amount of smooth muscle bundles between the groups of cells (Fig. 10). Examining the series it was impossible to escape the conclusion

---

EXPLANATION OF PLATES

FIG. 1.—Small-celled adenoma. H. and E. × 230.
FIG. 2.—Papillary foam-celled adenoma. H. and E. × 230.
FIG. 3.—Foam-celled adenoma with calcified nodule. H. and E. × 230.
FIG. 4.—Solid foam-celled adenoma. H. and E. × 230.
FIG. 5.—Eosinophilic adenoma. H. and E. × 230.
FIG. 6.—Adrenal rest partly outside and partly beneath the renal capsule. Note the included fat cells. H. and E. × 40.
FIG. 7.—Subcapsular adrenal rest. H. and E. × 50.
FIG. 8.—Pure lipoma. H. and E. × 50.
FIG. 9.—Subcapsular leiomyoma. H. and E. × 50.
FIG. 10.—Mixed leiomyo-lipoma. H. and E. × 50.
FIG. 11.—Fibrous nodule with many fibrocytes. H. and E. × 50.
FIG. 12.—Fibrous nodule with fine fibres running in all directions. H. and E. × 50.
FIG. 13.—Fibrous nodule with tubules running through. H. and E. × 50.
FIG. 14.—Fibrous nodule with hyaline. Irregular tubes of hyaline surround atrophic collecting tubules. H. and E. × 50.

1

2

3

4

5

6

Reese and Winstanley.

7

8

9

10

Reese and Winstanley.

11

12

13

14

that these three types were varieties of a single species of lesion with every gradation between them ; where a single kidney bore more than one, they often differed greatly in fat or muscle content. Van Gieson staining of the muscular elements showed a few collagen fibres but the tissue was predominantly muscular.

*Discussion*

Albrecht (1904) gave the word "Hamartoma" to a tumour-like malformation in which there was nothing more than an abnormal mixture of the normal tissue components of the organ. The abnormality might be in quantity or in dislocation or in respect of the grade of developmental maturity, or in all three of these respects. The small tumour-like masses we have described give no appearances of being independent neoplastic growths and they appear to fit Albrecht's definition of hamartoma. The tissues are a mixture of fat, smooth muscle and occasionally vessels. They appear to have been dislocated from the renal capsule or perhaps the pelvis. Smooth muscle is found in foetal but not in adult renal capsule so that an abnormality of developmental maturity must be the reason for its presence here. Unfortunately we have only one case appearing before middle age but large tumours of fat and muscle have been reported in children. Cottrell and Heckel (1954) described a baby girl of 3 months with such a tumour 5 × 2 cm. in size. A very large leiomyoma in a girl of 15 was described by Gordon, Kimmelstiel and Cabell (1939), who give a very good review of the literature. This incidence of such lesions in the young supports the idea of their being hamartomas, or in the case of the larger tumours, neoplasms arising in a hamartoma. Their presence in tuberose sclerosis (Hulse and Palik, 1951) also supports this view as in this disease such abnormalities are present in other organs. A finding which we are quite at a loss to explain is the much greater frequency in our series of this lesion in women. It is not sufficient to suggest that lipomas are mere adipose infiltrations and that women are obese more frequently than men. Pure myomas and mixed tumours were also much more frequent in women.

### *Medullary Fibrous Nodules*

A total of 159 fibrous nodules of the medulla were found in 78 of the kidneys examined. One kidney had 8 such nodules, 6 kidneys had 5, 7 had 4, 8 and 3, 13 had 2 and in 43 kidneys only a single nodule was found. The age and sex distribution is set out in Table VI.

TABLE VI.—*The Age and Sex Distribution of Medullary Fibrous Nodules*

| | 0–10 | 11–20 | 21–30 | 31–40 | 41–50 | 51–60 | 61–70 | 71–80 | 81–90 | 91–100 |
|---|---|---|---|---|---|---|---|---|---|---|
| Male cases . . | 0 | 0 | 1 | 1 | 3 | 9 | 11 | 15 | 5 | 0 |
| Female cases . . | 0 | 0 | 0 | 0 | 1 | 3 | 5 | 15 | 9 | 0 |
| Total cases . | 0 | 0 | 1 | 1 | 4 | 12 | 16 | 30 | 14 | 0 |
| Total lesions . | 0 | 0 | 1 | 2 | 8 | 21 | 42 | 55 | 30 | 0 |

The largest lesions, of which there were 17, were up to 0·3 cm. in diameter. There were 36 nodules of 0·2 or 0·25 cm., 91 of 0·1 or 0·15 cm. and 15 which were less than 0·1 cm. diameter. A number of the smaller lesions might have been lost or overlooked. These nodules were usually spherical in shape, occasionally oval. They consisted of fine fibrous tissue with a varying number of fibrocytes (Fig. 11). The fibres showed a " ball of twine " arrangement unlike the parallel fibres of the normal medullary connective tissue (Fig. 12). Most of the lesions had a few tubules running through them (Fig. 13). Although the majority of the nodules appeared to the naked eye as white, clearly defined tumours, there was no sharp edge to them when they were examined microscopically and they were never encapsulated. They did not contain smooth muscle and the occasional myoma of the medulla was easily distinguished. One-third of the nodules showed areas of a curious hyaline change in the connective tissue. The hyaline material stained a uniform bright pink with eosin and failed to give a red colour with Van Gieson. It was arranged in irregular streaks or in rounded tubes (Fig. 14).but it was obviously changed collagen and not thickened basement membrane because typical collagen fibres could be seen entering or leaving hyaline masses. In non-hyaline areas the collagen fibres formed around the remains of tubules so it was not remarkable that when collagen became hyaline it would take up their shape. In a few of the nodules, the ground substance between the fibres was mucoid and stained bluish with haematoxylin, an appearance often common to several nodules in the same kidney.

*Discussion*

These nodules were first described by Virchow (1863), who regarded them as inflammatory in origin. He said that there existed a circumscribed *nephritis interstitialis tuberosa* which was focal and gave place to fibrous tumours formed by progressive hyperplasia of the interstitial tissue, at the same time the collecting tubules contained in these areas underwent atrophy little by little. This view was questioned by Albrecht (1904), who said that there was no doubt that they were genuine hamartomas which differed from the normal only in the presence of an abnormal amount of connective tissue having a typical arrangement. The regular disposition of the tubules made it impossible to regard them as tumours in the strict sense, the connective tissue was laid down in a regular relation to the tubules, there was no sharp edge to the fibroma and no evidence of growth after completion of the growth of the kidney. He proposed, therefore, the name *hamartoma fibro-canaliculare renis* for these formations. Genewein (1905), who reviewed the literature on these lesions, followed Albrecht and called them hamartomas. The only recent study is that of Zangemeister (1936), who cut 99 pairs of kidneys on a ham slicer, 11 from the newborn and 9 or 10 from each subsequent decade. He found that 56 per cent of men and 60 per cent of women bore these lesions. The youngest was 18 years old and there was a constant increase with age, with an incidence of 80 per cent in the fifth decade. He distinguished three stages in the development of a nodule. Young lesions were rich in cells with a fine fibrillary skein-like arrangement of the collagen. Nodules in the second stage were moderately rich in cells with coarse fibres and perhaps a little hyaline, while old lesions were poor in cells, the connective tissue was split up by fissures and hyaline was abundant. He was able to show that the younger cases tended to have young lesions and the older cases old lesions. He concluded that the fibroma of the renal medulla was not a hamartoma in Albrecht's sense but was a fibrosis dependent on the generally increased tendency to tumour formation as age advanced. He compared it to verruca senilis or mucous polyp of the intestine.

These masses show little tendency to growth and do not appear to give rise to neoplasms. The large tumour described by Clar (1933) at the upper pole of the kidney in a man of 30 appeared to be a myoma and the hilar tumour in a man of 38 reported by Kretchmer (1932) a neurofibroma. There is no definite relationship between old infarcts or old pyelonephritis and these lesions. We have found no evidence of an inflammatory origin postulated by Virchow and we are inclined to favour the view of Zangmeister, comparing them perhaps to keloids rather than to polyps.

### SUMMARY

More than 200 kidneys removed from routine necropsies were cut on a ham slicer. All small nodules found were examined microscopically and with a few exceptions they fell into one of four categories. Suprarenal rests and small adenomas were found in the cortex, small fibrous lesions in the medulla, and in either cortex or medulla there were a number of lesions consisting of adipose tissue, smooth muscle or a mixture of these.

The suprarenal rests were regarded as ectopic suprarenal cortical tissue and were always on the surface of the organ. They were present in 5 per cent of the kidneys examined.

The cortical adenomas became commoner as age advanced. When many were present in a single organ there was usually severe scarring as well, but their relation to scars was inconstant. They were considered as neoplastic in origin though not actively growing and their relationship to hypernephromas was discussed.

Fibrous lesions of the medulla were also more common in the elderly. For this reason they were not considered to be hamartomas but focal overgrowths of fibrous tissue, perhaps like keloids, in response to an unknown stimulus.

Leiomyomas, lipomas and mixtures of these were all regarded as variants of the same lesion, a hamartoma. They were present at all ages and for some reason were commoner in women than in men.

We are indebted to Professor G. J. Cunningham for helpful advice and criticism, to Mr. S. Capon for technical assistance, and to Mr. E. B. Brain for the photomicrographs.

### REFERENCES

ALBRECHT, P.—(1904) *Verh. dtsch. path. Ges.*, **7**, 153.
APITZ, K.—(1943) *Virchows Arch.*, **311**, 285.
BOTSZTEJN, C. AND ZOLLINGER, H. U.—(1948) *Oncologia*, **1**, 165.
CLAR, F.—(1933) *Z. Urol.*, **27**, 477.
COTTRELL, T. L. C. AND HECKEL, N. J.—(1954) *J. Pediat.*, **45**, 206.
EWING, J.—(1928) 'Neoplastic Diseases', 3rd Edition. Philadelphia (W. B. Saunders & Co.), p. 782.
FRANKS, L. M.—(1954) *Ann. R. Coll. Surg. Engl.*, **15**, 236.
GENEWEIN, F.—(1905) *Z. Heilk.* (path. Anat.), **26**, 430.
GLYNN, E. E.—(1912) *Quart. J. Med.*, **5**, 157.
GORDON, M. P., KIMMELSTIEL, P. AND CABELL, C. L.—(1939) *J. Urol.*, **42**, 507.
GRAWITZ, P.—(1883) *Virchows Arch.*, **93**, 39.
HULSE, C. A. AND PALIK, E. E.—(1951) *J. Urol.*, **66**, 506.
KRETCHMER, H. L.—(1932) *Surg. Gynec. Obstet.*, **54**, 524.
NEWCOMB, W. D.—(1936) *Proc. R. Soc. Med.*, **30**, 113.

NICHOLSON, G. W. DE P.—(1909) *J. Path. Bact.*, **13**, 382.—(1950) ' Studies on Tumour Formation '. London (Butterworth), p. 104.
RAEBURN, C. AND SPENCER, H.—(1953) *Thorax*, **8**, 1.
SEMPRONJ, A. AND MORELLI, E.—(1939) *Amer. J. Cancer*, **35**, 534.
STOERK, O.—(1908) *Beitr. path. Anat.*, **43**, 393.
SUDECK, P.—(1893) *Virchows Arch.*, **133**, 405.
TRINKLE, A. J.—(1936) *Amer. J. Cancer*, **27**, 676.
VIRCHOW, R.—(1863) ' Die krankhaften Geschwülste '. Berlin (August Hirschwald), vol. 1, p. 333.
WILLIS, R. A.—(1953) ' Pathology of Tumours ', 2nd Edition. London (Butterworth), p. 456.
ZANGEMEISTER, W.—(1936) *Beitr. path. Anat.*, **97**, 142.
ZOLLINGER, H. U.—(1953) *Virchows Arch.*, **323**, 694.